# A clinical and molecular epidemiological survey of hepatitis C in Blantyre, Malawi, suggests a historic mechanism of transmission

**DOI:** 10.1111/jvh.13646

**Published:** 2022-02-09

**Authors:** Alexander J. Stockdale, Benno Kreuels, Isaac T. Shawa, James E. Meiring, Deus Thindwa, Niza M. Silungwe, Karen Chetcuti, Elizabeth Joekes, Maurice Mbewe, Blessings Mbale, Pratiksha Patel, Rabson Kachala, Priyanka D. Patel, Jane Malewa, Peter Finch, Chris Davis, Rajiv Shah, Lily Tong, Ana da Silva Filipe, Emma C. Thomson, Anna Maria Geretti, Melita A. Gordon

**Affiliations:** ^1^ Malawi‐Liverpool‐Wellcome Trust Programme Blantyre Malawi; ^2^ Institute of Infection, Veterinary and Ecological Sciences University of Liverpool Liverpool UK; ^3^ Department of Tropical Medicine Bernhard Nocht Institute for Tropical Medicine and 1st Department of Medicine University Medical Centre Hamburg‐Eppendorf, Hamburg Germany; ^4^ Kamuzu University of Health Sciences Blantyre Malawi; ^5^ Oxford Vaccine Group Department of Paediatrics University of Oxford Oxford UK; ^6^ Department of Infectious Disease Epidemiology London School of Hygiene and Tropical Medicine London UK; ^7^ Malawi Ministry of Health Capitol Hill, Lilongwe Malawi; ^8^ Department of Medicine Queen Elizabeth Central Hospital Blantyre Malawi; ^9^ MRC ‐ University of Glasgow Centre for Virus Research University of Glasgow Glasgow UK; ^10^ Department of Infectious Diseases Fondazione PTV Faculty of Medicine University of Rome Tor Vergata Rome Italy

**Keywords:** Africa, cirrhosis, epidemiology, hepatitis C, Malawi, South of the Sahara

## Abstract

Hepatitis C virus (HCV) is a leading cause of liver disease worldwide. There are no previous representative community HCV prevalence studies from Southern Africa, and limited genotypic data. Epidemiological data are required to inform an effective public health response. We conducted a household census‐based random sampling serological survey, and a prospective hospital‐based study of patients with cirrhosis and hepatocellular carcinoma (HCC) in Blantyre, Malawi. We tested participants with an HCV antigen/antibody ELISA (Monolisa, Bio‐Rad), confirmed with PCR (GeneXpert, Cepheid) and used line immunoassay (Inno‐LIA, Fujiribio) for RNA‐negative participants. We did target‐enrichment whole‐genome HCV sequencing (NextSeq, Illumina). Among 96,386 censused individuals, we randomly selected 1661 people aged ≥16 years. Population‐standardized HCV RNA prevalence was 0.2% (95% CI 0.1–0.5). Among 236 patients with cirrhosis and HCC, HCV RNA prevalence was 1.9% and 5.0%, respectively. Mapping showed that HCV RNA+ patients were from peri‐urban areas surrounding Blantyre. Community and hospital HCV RNA+ participants were older than comparator HCV RNA‐negative populations (median 53 vs 30 years for community, *p *= 0.01 and 68 vs 40 years for cirrhosis/HCC, *p *< 0.001). Endemic HCV genotypes (*n* = 10) were 4v (50%), 4r (30%) and 4w (10%). In this first census‐based community serological study in Southern Africa, HCV was uncommon in the general population, was centred on peri‐urban regions and was attributable for <5% of liver disease. HCV infection was observed only among older people, suggesting a historic mechanism of transmission. Genotype 4r, which has been associated with treatment failure with ledipasvir and daclatasvir, is endemic.

**Abbreviations**
HCC: hepatocellular carcinoma
HCV: Hepatitis C virus
IQR: interquartile range
PAF: population‐attributable fraction
PUF: population‐unattributable fraction
QECH: Queen Elizabeth Central Hospital
ULN: upper limit of normal

## 1 INTRODUCTION

In 2016, the World Health Assembly approved targets to reduce mortality from viral hepatitis by 65% and reduce incidence by 90% by 2030.^1^ To achieve these goals, accurate epidemiological data are necessary to inform an effective public health response. In sub‐Saharan Africa, an estimated 1% of the population, representing 11 million people, have hepatitis C virus (HCV) infection. These estimates, however, are largely derived from convenience sampling, with a paucity of epidemiologically representative data, particularly in Southern Africa.^1^ HCV prevalence estimates were revised downward as a result of recent data from West Africa showing lower prevalence relative to previous samples and with the inclusion of confirmatory HCV RNA testing data.^2^ Prevalence estimates based on anti‐HCV antibody testing have been associated with overestimation due to low specificity.^2^, ^3^, ^4^ In a pooled analysis of 35 studies from sub‐Saharan Africa, HCV RNA PCR testing confirmed active infection among only 51% of anti‐HCV positive people.^5^

Hepatitis C virus treatment programmes are not yet widely available in the region, although there are unprecedented opportunities for disease control with the advent of point‐of‐care serological and molecular tests and direct‐acting pan‐genotypic antivirals. Achieving HCV elimination will first require accurate estimate of disease burden, elucidation of key populations at risk of infection and understanding of locally important specific risk factors for transmission.^6^ A recent systematic review did not identify any high‐quality general population HCV epidemiological data for Southern Africa, and included only a single study from the region, from South Africa, which tested a convenience sample from patients attending clinics.^2^

We addressed these research needs first by estimating HCV prevalence in the general population, by conducting a single‐stage random probability sampling household serosurvey based on a demographic census, in Blantyre, Malawi. Second, we prospectively recruited patients with cirrhosis and hepatocellular carcinoma presenting to a tertiary referral hospital in Blantyre, to estimate the prevalence and attributable fraction of hepatitis C to liver disease. Third, we sequenced whole HCV genomes from viraemic patients to characterize the molecular epidemiology of HCV in a region with very limited prior genotypic data. Finally, we conducted a systematic review of existing epidemiological data on hepatitis C to characterize the existing evidence on HCV prevalence in the general population in Southern Africa.

## 2 MATERIALS AND METHODS

### 2.1 Census and serological survey

Individuals aged ≥16 years were selected using single‐stage random sampling from a recent full‐population demographic census of an urban population of 96,386 and invited to participate in a serosurvey conducted between 14 December 2016 and 12 April 2018 as part of a co‐incident ongoing typhoid epidemiology study (Strategic Typhoid Alliance across Africa and Asia) in Ndirande, Blantyre.^7^ If a randomly selected individual could not be located or did not consent to participation, another household member aged >16 years was requested to participate, or secondarily, a replacement was selected using further randomization from the census. Demographic, educational, marital, and occupational data were recorded. Venous EDTA samples were collected in participants’ households by research nurses, stored in cool boxes and transported to the study laboratory from the field. Plasma samples were separated by centrifugation and stored at −80°C.

### 2.2 Clinical evaluation of liver disease

We returned to households of serosurvey participants who tested positive for HCV Ag‐Ab by enzyme immunoassay (ELISA) and invited them to participate in a clinical and virological evaluation. We additionally randomly selected a sample of 65 unmatched community controls aged >16 years from the serosurvey population and negative for HCV Ag‐Ab and HBsAg to measure liver stiffness normal range. HIV testing was offered to participants using rapid diagnostic tests in accordance with national guidelines using Determine HIV (Alere, South Africa) and confirmatory testing with Uni‐Gold HIV (Trinity Biotech, Ireland). We assessed liver stiffness using transient elastography in the right mid‐axillary intercostal space after fasting for >3 h (FibroScan 430 Mini, Echosens, France).^8^ Reliability criteria were IQR/median <0.3 if median LSM >7.1 kPa.^9^

### 2.3 Sample size calculation

Based on previous estimates of anti‐HCV prevalence from Malawi in the preceding 20 years from convenience samples ranging from 0.5% to 10%,^10^ we estimated sample size using a conservative prior prevalence estimate of 10% with 1.5% precision with a 95% confidence interval, giving a sample size requirement of 1537 for the community serosurvey (Appendix 1). No previous data were available describing the normal population distribution of liver stiffness among healthy individuals in sub‐Saharan Africa, nor among community patients with hepatitis C infection. Using estimates from a study of people with hepatitis C infection who inject drugs in Tanzania,^11^ and a normal population reference from a systematic review of 26 cohort studies,^12^ our calculated sample size requirement for detecting a difference in the predicted mean liver stiffness measurement between patients with hepatitis C and healthy population controls was 64 in each group (Appendix 1).

### 2.4 Hospital‐based study

We recruited consecutive patients aged ≥16 years presenting at the Queen Elizabeth Central Hospital (QECH) in Blantyre, Malawi, during an 18‐month period (November 2017 to April 2019). QECH acts as a referral hospital for the southern region of Malawi. Study nurses from Monday to Friday systematically reviewed clinical notes of all newly admitted patients to medical and surgical wards and attendees at medical clinics and the endoscopy unit. Study nurses applied screening criteria for features of cirrhosis and HCC comprising any of: (i) signs of chronic liver disease: jaundice, ascites, splenomegaly, spider telangiectasia, gynecomastia, dilated veins of the lower abdominal wall, digital clubbing or asterixis; or (ii) upper gastrointestinal bleeding from oesophageal or gastric varices demonstrated on upper GI endoscopy; or (iii) patients with suspected hepatocellular carcinoma: having an abdominal mass in the right upper quadrant and/or a hepatic lesion identified on radiological imaging. Patients were eligible for participation if liver stiffness was ≥11 kPa on transient elastography, or if there was a hepatic mass consistent with HCC following a study ultrasound examination. Liver stiffness was measured in the right mid‐axillary intercostal space by transient elastography after fasting for >3 h (FibroScan 430 Mini, Echosens, France).^8^ Reliability was assessed by an interquartile range (IQR)/median <0.3.^9^ Exclusion criteria were pregnancy or patients with potential reasons for false elevations in liver stiffness, comprising: an absence of evidence of ultrasound features of cirrhosis together with (i) acute hepatitis with ALT elevation >2× the upper limited of normal (ULN) or (ii) biliary dilatation or obstruction or (iii) cardiac failure defined as clinical symptoms and signs of heart failure (orthopnoea, paroxysmal nocturnal dyspnoea, elevated jugular venous pressure, third heart sound, bibasal lung crepitations, hepatomegaly, response to diuretic therapy) or ultrasound evidence of a dilated inferior vena cava >2.1 cm measured 1cm inferior to the cavoatrial junction with dilated hepatic veins^13^ Ultrasound evidence of cirrhosis was defined as a coarse liver parenchymal echotexture; an irregular, nodular liver surface; narrowed or tortuous hepatic veins; and a small liver with hypertrophy of caudate lobe, the presence of splenic varices or the presence of ascites or splenomegaly (>12.7 cm) without an alternative identified cause.^14^, ^15^, ^16^ We used an ultrasound‐based definition of HCC, reflecting that contrast‐enhanced computed tomography or magnetic resonance imaging was not locally available and histological confirmation was rarely possible. Ultrasound investigations were conducted using a standardized B‐mode protocol by a member of the study team (AS, KC, EJ or BK) with a portable ultrasound machine (CTS 7700, SIUI, China), using a 4.2‐MHz curvilinear probe. HCC was defined as an intrahepatic mass of at least 1 cm with an ultrasound appearance suggestive of HCC, including mass effect on surrounding structures or macrovascular invasion involving the portal vein. For smaller or indeterminate lesions, repeat ultrasound was conducted at intervals of 1–2 months and enlarging and evolving lesions were considered consistent with HCC. Consultant radiologists experienced in the diagnosis of liver disease (KC, EJ) reviewed imaging of uncertain lesions and/or performed follow‐up ultrasound examinations.

### 2.5 Laboratory investigations

A combined hepatitis C virus (HCV) antigen and antibody ELISA (HCV Ag‐Ab ULTRA V2, Bio‐Rad, France) was used as an initial test, in accordance with the manufacturer's instructions. All positive and indeterminate samples (sample‐to‐cut‐off ratio (S/CO) >0.9) were retested in duplicate. Positives were tested with HCV RNA PCR (GeneXpert HCV, Cepheid, South Africa). We tested all HCV Ag‐Ab+/HCV RNA‐ patients with available samples using a line immunoassay (Inno‐LIA HCV score, Fujirebio, Japan). We tested for HBsAg using Monolisa HBsAg‐Ultra (Bio‐Rad), and positive and indeterminate samples (S/CO >0.9) were repeated in duplicate.

### 2.6 HCV sequencing and bioinformatic analysis

Hepatitis C virus whole‐genome sequencing was performed for all HCV RNA‐positive participants using a previously described method for target enrichment using probe capture, followed by next‐generation sequencing using the NextSeq 550 platform (Illumina, USA) with 150‐bp paired read length.^17^ Tanoti (http://www.bioinformatics.cvr.ac.uk/tanoti.php) was used to align unmapped reads against HCV reference sequences from the International Committee on Taxonomy of Viruses (https://talk.ictvonline.org/).^18^ HCV GLUE was used to identify the subgenotype and resistance‐associated mutations, and repeat alignment was made using the closest subtype reference sequence.^19^ Sequence alignment was performed using MAFFT with L‐INS‐I against near‐full‐length genotype 4 genomes in the HCV Glue reference repository. Uncorrected p‐distance from closest reference sequences was calculated using MEGA 10.1.6. JModeltest2 was used to identify the optimal phylogenetic model.^20^ A phylogenetic tree was constructed with full‐length genotype 4 sequences Randomized axelerated maximum likelihood, using a maximum‐likelihood model with general time‐reversible model with gamma distribution with invariant sites with nearest‐neighbour‐interchange heuristics with 1000 bootstrap replications.^21^

### 2.7 Ethical review

Ethical permission to conduct this study was obtained from the National Health Sciences Research Committee of Malawi (16/11/1698 and 15/5/1599) and the University of Liverpool Research Ethics Committee (reference 1954). Census informants and participants in the serological survey, community evaluation and hospital‐based study provided written informed consent. The study protocol conformed to the ethical guidelines of the 1975 Declaration of Helsinki.

### 2.8 Systematic review of existing data

We systematically searched for previous epidemiological data of hepatitis C prevalence in the general population in Southern Africa (full details are described in Appendix 2). Searches were conducted in PubMed, with no language or date restriction, for synonyms of hepatitis C or diagnostic tests and the countries in the African Union Southern Region. We included studies which reported anti‐HCV antibodies or HCV RNA prevalence among a sample of the general population using an epidemiologically representative sampling method.

### 2.9 Statistical analysis

We estimated population‐standardized HCV prevalence using post‐stratification iterative proportional fitting using 5‐year age groups and sex from the census to adjust prevalence estimates to reflect the population age and sex distribution.^22^ We calculated the population‐attributable fraction (PAF) of HCV to liver disease by comparing the prevalence of HCV RNA among patients with cirrhosis and HCC to the community prevalence. Following logistic regression, a post‐estimation predictive margin of response was estimated for a modelled scenario with HCV RNA prevalence of zero. The ratio between the logit of the baseline likelihood and the zero exposure scenario was calculated, representing the population‐unattributable fraction (PUF), and PAF was calculated using (1‐ PUF). PAF calculations were implemented using the *punafcc* package.^23^ Association between HCV RNA‐positive participants and explanatory variables were assessed by comparison with the HCV RNA‐negative population using Fisher's exact, chi‐squared or Wilcoxon rank‐sum test as appropriate, and using a binomial logistic regression model. We selected variables for inclusion in the model based on *a priori* clinical relevance and to optimize model fit using the Bayesian information criteria. We modelled the geographic incidence of cases of cirrhosis and HCC per 10,000 population using data from the National Population Census 2018.^24^ Analyses were conducted using Stata 16.1 (StataCorp, USA) and ArcGIS Pro 2.8 (ESRI, USA).

## 3 RESULTS

### 3.1 Community serological survey

**FIGURE 1:**
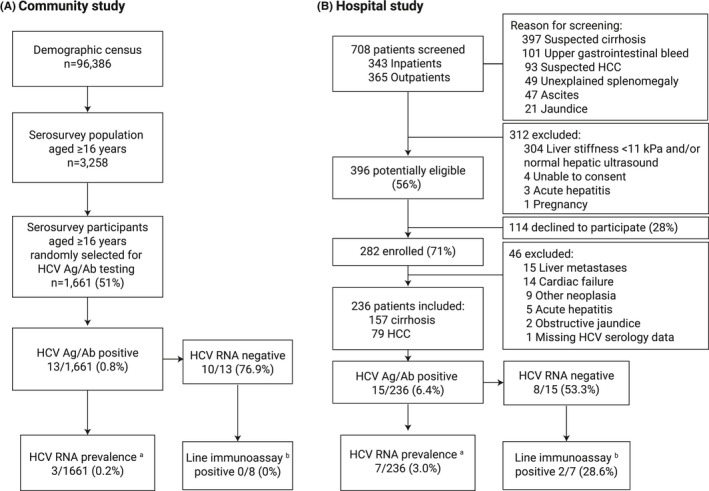
Flow chart of study recruitment from community (A) and hospital study (B). ^a^HCV RNA was tested among HCV Ag‐Ab–positive individuals. ^b^Line immunoassay was tested among HCV Ag‐Ab–positive/ HCV RNA‐negative individuals with available samples, comprising 8 of 10 (80%) eligible community patients and 7 of 8 inpatients (88%). Abbreviations: HCV, hepatitis C virus; Ag‐Ab, antigen/antibody; RNA, ribonucleic acid; HCC, hepatocellular carcinoma

We randomly selected a total of 1661 participants from the community census aged ≥16 years for HCV testing, with median age 30 years (interquartile range (IQR) 22, 39); 52.7% (876/1661) were female (Figure 1). In the National Population Census 2018, median age was 30 years (IQR 23, 43) and 52.3% were female among individuals ≥16 years.^24^ The serosurvey population thus closely represented the census age and sex distribution (Appendix 3). HCV Ag‐Ab was positive in 13 of 1661 patients, and the population‐standardized HCV Ag‐Ab prevalence was 0.8% (95% CI: 0.5–1.4). HCV RNA was detected in 3/13 (23.1% (95% CI 8.2–50.3)) with HCV RNA concentrations of 5.94, 6.63 and 7.09 log_10_ IU/mL, respectively. Population‐standardized HCV RNA prevalence was 0.2% (95% CI 0.1–0.5). Among 8 of 10 individuals with available samples, all Ag‐Ab+/RNA‐ participants were negative for HCV antibodies by line immunoassay.

The three community HCV RNA‐positive individuals (median age 53 years (IQR 46, 55)) were significantly older than the HCV RNA‐negative community serosurvey population (median 30 years (IQR 22, 39, *p *= 0.01), and two (67%) were female. All were negative for HBsAg. One (33%) was HIV‐positive and on antiretroviral therapy. None had clinical signs of chronic liver disease. Liver stiffness was, however, raised in all three at 7.1, 7.8 and 8.3 kPa, respectively, relative to the community control sample (*n* = 65) whose median liver stiffness was 4.5kPa (IQR 3.5, 5.3), *p *= 0.002.

### 3.2 Hospital cohort study

Over an 18‐month period, we screened 708 patients with suspected liver disease and recruited 236 consecutive patients attending the hospital and meeting study definitions for cirrhosis (*n* = 157) or HCC (*n* = 79) (Figure 1). Participant median age was 40 years (IQR 34, 51), and 143 (60.3%) were male. Overall, 10 of 157 (6.4%) patients with cirrhosis and 5 of 79 patients with HCC (6.3%) were HCV Ag‐Ab–positive, and HCV RNA was positive in 3 of 10 and 4 of 5 patients, respectively, for an HCV RNA prevalence of 1.9% (95% CI 0.4–5.5) among patients with cirrhosis and 5.1% (95% CI 1.4–12.5) in patients with HCC. Line immunoassay was positive for HCV antibodies in 2 of 7 (28.7%) patients who were HCV Ag/Ab–positive and RNA–negative. The population‐attributable fraction for hepatitis C, relative to the community population, for cirrhosis was 1.7% (95% CI −0.4 to 3.9) and for HCC was 4.9% (95% CI 0.0–9.6).

The seven HCV RNA+ patients’ median age was 69 years (IQR 64, 73), and they were significantly older than HCV RNA‐ patients with cirrhosis or HCC (median 40 years (IQR 33, 50)), *p *< 0.001 (Figure 3). All 7 HCV RNA+ patients were hepatitis B surface antigen (HBsAg)‐ and HIV‐negative. HCV RNA‐positive patients reported a median symptom duration of 4 months, with the majority presenting with abdominal pain or ascites. Clinical signs of chronic liver disease were observed in 6 of 7 (86%) patients, with cachexia and ascites predominating (Appendix 4).

Positive predictive value of a positive HCV Ag‐Ab test for HCV RNA+ viraemic infection was 23.1% (95% CI 13.9–35.8) among community participants and 46.7% (95% CI 30.7–63.4) for patients with cirrhosis or HCC. Among HCV RNA+ participants, HCV Ag‐Ab ELISA sample‐to‐cut‐off ratios (S/CO) were significantly higher with a mean S/CO of 13.4 (95% CI 11.9–14.9) relative to 3.8 (95% CI 0.8–6.9) among HCV RNA‐ individuals, *p *< 0.001 (Appendix 5). All HCV RNA+ individuals had a S/CO value ≥9.

### 3.3 Risk factors for HCV infection

**FIGURE 2:**
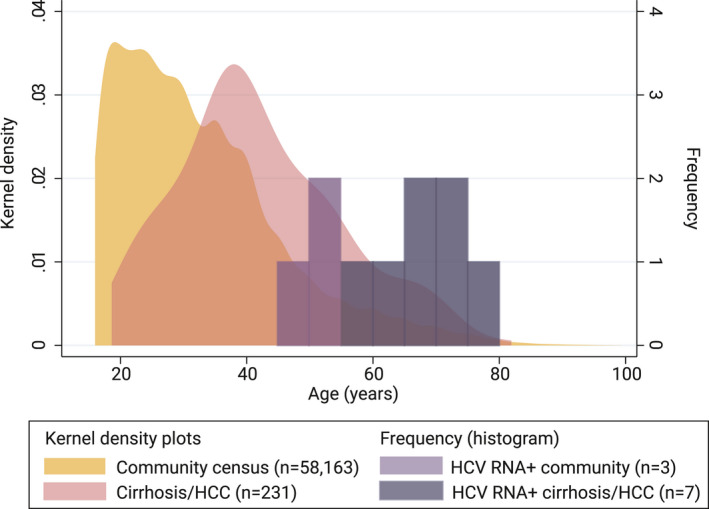
Age distribution of community census, patients with cirrhosis and HCC and HCV RNA‐positive patients from the community and hospital studies^a^. ^a^The kernel density plot for the census includes people aged ≥16 years, amounting to 58,163/96,834 of the total census population. The kernel density plot of 231 cirrhosis/HCC patients from the hospital study excludes HCV RNA‐positive patients, who are presented in the dark purple histogram

Hepatitis C virus RNA+ individuals from the community and hospital study were significantly older than the community HCV RNA‐ population (Figure 2) and were less likely to have undergone secondary education, and were more likely to be divorced or separated, by univariable analysis (Table 1). After adjustment for age, incorporating a quadratic term, single or divorced or separated individuals were significantly more likely to be HCV RNA+, relative to married individuals.

**TABLE 1:** Characteristics of HCV RNA+ individuals relative to community HCV RNA‐ controls: binomial logistic regression model

Characteristics	HCV RNA+ individuals *n* = 10	HCV RNA− community controls *n* = 1658	Univariable associations with HCV RNA+		Multivariable model	
			Odds ratio (95% CI)	*p* value	Odds ratio (95% CI)	*p* value
Age (years); models: per year	65 (59, 71)	30 (22, 39)	1.1 (1.1–1.2)	<0.001	2.7 (1.1–6.8)†	0.03
Sex, female	6 (60)	874 (52.7)	1.4 (0.4 – 4.8)	0.65		
Marital status				0.12		0.002
Married	5 (50)	895 (58.1)	Reference		Reference	
Single	2 (20)	533 (34.6)	0.7 (0.1–3.5)		28.9 (2.2–377.7)	
Divorced/separated	2 (20)	43 (2.8)	8.3 (1.6–44.1)		46.4 (3.7–579.2)	
Widowed	1 (10)	69 (4.5)	2.6 (0.3–22.5)		0.5 (0.1–4.4)	
Occupational status				0.62		
Unemployed	5 (50)	438 (28)	Reference			
Self‐employed	3 (30)	471 (30)	0.6 (0.1–2.3)			
Employed	2 (20)	345 (22)	0.5 (0.1–2.6)			
Education				0.005		
None	2 (20)	27 (2)	Reference			
Primary	5 (50)	487 (31)	0.1(0.03–0.8)			
High school	1 (10)	946 (60)	0.01 (0.01–0.2)			
Vocational	1 (10)	62 (4)	0.2 (0.02–2.5)			
University	1 (10)	55 (3)	0.3 (0.02–2.8)			

^†^ A quadratic term for age was included in the multivariable model.

**FIGURE 3:**
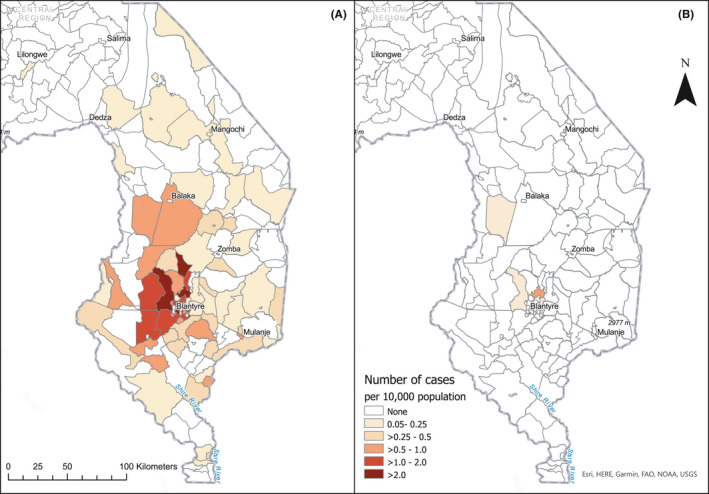
Residence of patients with HCC and cirrhosis referred to Queen Elizabeth Central Hospital over an 18‐month period, per 10,000 population. (A) HCV RNA‐negative patients; (B) HCV RNA‐positive patients^a^. ^a^Population data for each traditional area are from the National Population Census 2018

Geographic mapping of residence of patients with liver disease showed that all but one HCV RNA+ individuals with cirrhosis or HCC population referred to the hospital were located within the Blantyre city region, whereas the remaining 229 HCV RNA‐negative patients were distributed broadly throughout southern region, with higher referral rates from the area surrounding Blantyre (Figure 3). Urban or peri‐urban residence was reported by 86% (6/7) of HCV RNA+ patients relative to 47% (108/229) of HCV RNA‐ cases, *p *= 0.06 (Fisher's exact test).

### 3.4 Molecular epidemiology of HCV

**FIGURE 4:**
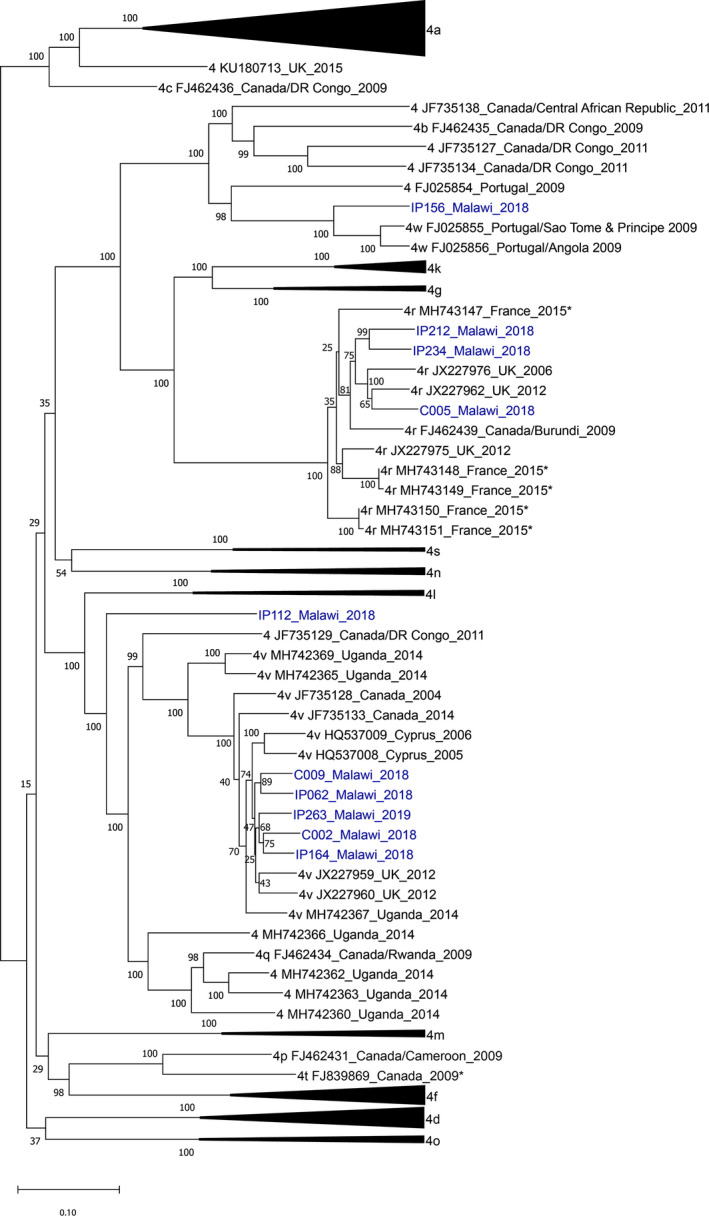
Maximum‐likelihood phylogenetic tree: hepatitis C sequences from Malawi aligned to 117 genotype 4 full‐genome sequences. *Sequence from an individual originally from sub‐Saharan Africa, where country data were not available. Maximum‐likelihood phylogenetic tree of 10 HCV sequences from Malawi with available full‐length genotype 4 sequences using a general time‐reversible model with gamma‐distributed rate among sites with invariant sites (GTR + G + I) with 1000 bootstrap replicates. Branch node numbers indicate bootstrap support. The tree with the highest log likelihood is shown. Sequences from subgenotypes 4a, d, f, k, l, n, o and s are collapsed. Novel sequences from Malawi from this study are highlighted in blue. Sequence labels indicate subgenotype, GenBank sequence number, country data and year of sequence acquisition. Abbreviations: UK, United Kingdom; DR, Congo Democratic Republic of Congo

Hepatitis C virus sequences containing the complete open reading frame were obtained from all 10 viraemic patients (3 from the community and 7 from the hospital setting). Mean read depth was 30,821 reads per nucleotide position, and mean coverage was 98%. All sequences were genotype 4. Maximum‐likelihood phylogenetic analysis with 117 characterized full‐length genotype 4 sequences showed that sequence subgenotypes were 4v (*n* = 5), 4r (*n* = 3) and 4w (*n* = 1). One sequence did not cluster with a subtype reference and was classified as an unassigned genotype 4 subtype (3 subtypes are required to define a new subgenotype)^25^ (Figure 4). The three 4r sequences most closely aligned with sequences from a patient from Burundi, and two sequences from the United Kingdom. The 4w sequence aligned with sequences from two Portuguese patients who lived in Sao Tome and Principe and Angola, respectively. The five 4v sequences aligned with sequences from Uganda, Cyprus and the United Kingdom. Pairwise distance analysis showed a high degree of diversity (Table 2). Analysis of the three 4r sequences showed distinct polymorphisms in NS5A at positions M28V (in two of three patients), and at L30V and L31V in all, which have been associated with resistance to ledipasvir and daclatasvir (Table 2).^26^

**TABLE 2:** Characteristics of HCV sequences: uncorrected pairwise distances from reference sequences and resistance‐associated substitutions: prevalence of predicted amino acid variants at non‐structural protein 5A (NS5A) positions^†^

Patient	Genotype	Closest reference sequence‡	*p*‐distance	NS5A amino acid positions (% of reads)			
				28	30	31	58
C005	4r	FJ462439	0.085	M (99)	R (100)	L (99)	P (100)
IP212	4r	FJ462439	0.081	V (99)	R (99)	L (99)	P (100)
IP234	4r	FJ462439	0.082	V (99)	R (100)	L (99)	P (100)
C009	4v	JX227959	0.061	‐	R (100)	‐	P (100)
C002	4v	JX227959	0.065				
IP062	4v	JX227959	0.059	‐	R (100)	‐	P (100)
IP164	4v	JX227959	0.061	M (66)	R (100)	M (100)	P (100)
IP263	4v	JX227959	0.060		R (100)	‐	P (100)
IP156	4w	FJ025855	0.103	‐	‐	‐	P (100)
IP112	4*	FJ462434	0.146	‐	R (85)	‐	P (100)

^†^ Patient numbers starting with ‘COM’ reflect community serosurvey participants; those starting ‘IP’ are patients with cirrhosis or HCC. Sequences are grouped by subtype. No Y93C/H/S variants were observed.

^‡^ GenBank accession number of International Committee of Taxonomy of Viruses HCV reference sequence with closest alignment.

### 3.5 Systematic review of existing HCV epidemiological data

A search using the PubMed database of countries included in the Southern Africa region identified 151 studies; 100 were excluded due to having no epidemiological data on HCV, or data outside the region (Appendix 2). Remaining studies were reported from convenience samples (32 studies), consisting of attendees of HIV clinics, antenatal clinics or blood donors, or represented populations at increased risk for bloodborne virus exposure (11 studies), the majority of whom were people who inject drugs (6). Of the remaining five studies, three were reported from hospital patients. Two studies were sampled from the general population but did not report an epidemiologically representative sampling method. No previous studies of HCV prevalence in the general population from Southern Africa using a representative sampling method were identified.

## 4 DISCUSSION

In this first census‐based community HCV prevalence using representative random sampling in Southern Africa, we found that in the general population, HCV was uncommon with a prevalence of 0.2%. Previous prevalence studies from the region have suffered from important biases that may limit sample representativeness. These include recruitment without a random sampling framework or using opportunistic sampling methods. We consulted three systematic reviews of HCV prevalence and performed an additional systematic search and did not identify any previous epidemiologically representative community prevalence studies for the region.^2^, ^5^, ^27^ Our sample used household recruitment, based on random sampling from a population census, and closely matched the national census age and sex distribution. Our estimated HCV prevalence rates were significantly lower than existing estimates for the wider region,^2^, ^5^ and our data suggest a concentrated HCV epidemic that is confined to older individuals and peri‐urban areas. If these data are confirmed with other representative population surveys from other sites in Southern Africa, the viraemic prevalence may be significantly lower than current estimates of 0.7%, representing 500,000 among the population of Southern Africa of 76 million.^2^ Analysis of the geographic distribution referred with liver disease to the regional centre showed that all but one HCV patient was from Blantyre City, relative to an urban distribution for less than half of remaining patients referred with liver disease of other causes. Our data suggest that current data that are largely convenience samples from urban populations may overestimate true prevalence, since the majority of individuals live in rural settings in this region.

In our study, the strongest specific risk factor associated with HCV infection was increasing age. All people diagnosed with HCV in both community and hospital cohorts were aged older than 45, and all HCV‐associated liver disease patients were older than 55 years, and significantly older than HCV‐negative control populations. This suggests that a historic risk factor for transmission, such as nosocomial transmission through reuse of needles or unscreened blood transfusion, was responsible. This finding is consistent with studies from Uganda^28^ and Cameroon^29^ where a similar cohort effect was observed. Although our sample size was calculated to estimate general population HCV prevalence with a precision of 1.5%, the lower‐than‐expected prevalence precluded in‐depth assessment of specific risk factors.

Additional prevalence surveys are now required among specific population groups at increased risk of HCV such as those who have received unscreened blood transfusions or injected treatments, men who have sex with men, people who inject drugs and commercial sex workers. Indeed, World Health Organization (WHO) guidelines recommend targeted screening for at‐risk populations where the general population prevalence of HCV is less than 2%.^30^ Estimates among convenience samples of HIV‐positive populations attending for routine HIV care in Malawi and recent data from Zambia, Mozambique, Uganda and Kenya similarly found low prevalence with anti‐HCV prevalence ranging from 0.2% to 1.1% and HCV RNA from 0% to 1%.^31^, ^32^, ^33^, ^34^

Reflecting the findings of the general population survey, we found that HCV was an uncommon cause of liver disease in the southern region of Malawi, with a population‐attributable fraction of less than 2% in patients with cirrhosis and 5% of HCC cases. Our study highlights the limitations of using convenience samples as proxies for community prevalence estimation. Innovations in patient sampling including the use of dried blood spot sampling to mitigate issues around cold storage and use of point‐of‐care oral fluid‐based antibody testing may facilitate future representative community‐level sampling.^35^, ^36^ Population‐level representative HIV viral load assessments represent a model and potential opportunity for performing combined viral hepatitis surveys.^37^

We observed that using HCV Ag‐Ab ELISA at the recommended sample‐to‐cut‐off (S/CO) threshold resulted in a high proportion of false‐positive results in this population, with a positive predictive value for viraemic infection of less than 25% in the community and less than 50% among inpatients. The HCV Inno‐LIA line immunoassay we used can differentiate past infection from a false‐positive result in the context of positive serology for Ag‐Ab+, but negative HCV RNA PCR. In the community study, all such individuals were all negative by line immunoassay; similarly, less than one third of such liver disease patients were positive. Our findings demonstrate a high rate of false‐positive Ag‐Ab results and add to evidence that epidemiological HCV estimates should not be based on serological tests without additional confirmatory nucleic acid amplification tests. Furthermore, we confirmed the finding that the magnitude of the ELISA S/CO ratio predicted HCV RNA positivity, similar to observations from HIV clinics in Ghana and among blood donors in Rwanda.^4^, ^38^ Our study adds to this evidence that using an increased S/CO ratio for serological assays threshold might reduce unnecessary confirmatory testing. The optimal threshold should be specific to the assay and population pre‐test probability of HCV RNA and should be validated for the specific setting where it is used.

The full‐length HCV genomes obtained in this study are the only available HCV sequences from Malawi and show that all belonged to genotype 4. Subgenotype 4r was observed in three patients from the community and hospital study. Previous data have suggested a geographic distribution of 4r among East and Central Africa, including Rwanda and the Democratic Republic of Congo. Our 4r sequences had L28 M/V, L30R and L31R mutations within the NS5A gene that have been associated with resistance to NS5A inhibitors and treatment failure with ledipasvir‐, velpatasvir‐ or daclatasvir‐based regimens in studies from Rwanda and in Europe.^26^, ^39^, ^40^ WHO treatment guidelines currently recommend empirical pan‐genotypic HCV treatment for genotype 4 using sofosbuvir with velpatasvir or daclatasvir for adults and sofosbuvir/ledipasvir for adolescents.^41^ Studies are required to evaluate the efficacy of empirical treatment regimens with locally prevalent genotypes, particularly describing the outcomes of treating 4r in Malawi.

In conclusion, in this representative census‐based community sample in Southern Africa, HCV infection was uncommon and lower than regional estimates based on convenience samples. We did not identify any prior epidemiologically representative community prevalence data from the region. In our hospital study, HCV was attributable to 5% of HCC and 2% of cirrhosis cases. HCV infection was observed only among people older than 45 years, suggesting a historic mechanism of transmission. All sequences were genotype 4, and subtype 4r, which has been associated with NS5A inhibitor resistance, is endemic. Representative sampling from specific risk groups in the region is now required.

## CONFLICT OF INTEREST

Prof. Geretti reports grants and personal fees from Roche Pharma Research & Early Development, grants and personal fees from ViiV Healthcare, personal fees from Janssen, and grants and personal fees from Gilead, outside the submitted work. The remaining authors report no competing interests.

## Supporting information

Supplementary Material
