# Supplementary material for: A clinical and molecular epidemiological survey of hepatitis C in Blantyre, Malawi, suggests a historic mechanism of transmission
Source: J Viral Hepat. 2022 Feb 9;29(4):252–62. doi: 10.1111/jvh.13646 (PMC9305194; doi:10.1111/jvh.13646)
Supplement: Supplementary file 1 — Supplementary Material [file JVH-29-252-s001.docx]

**Supplementary Appendix 1: Sample size calculations**

| **Population** | **Statistic** | **Reference** |
| --- | --- | --- |
| Estimated adult anti-HCV prevalence in Malawi | 0.5-10% | ^1^ |
| Census population size (for finite population) | 97,386 | Study census |
| Estimated mean normal reference range for liver stiffness in a healthy population | 4.68  (95% confidence interval 4.64-4.73);  Standard deviation 1.43 | ^2^ |
| Estimated mean liver stiffness among patients with hepatitis C in sub-Saharan Africa (cohort study of people who inject drugs in Tanzania) | Median= 5.4 (Interquartile range 4.4-6.5)  (sample size= 153)  Estimated mean (SD)= 5.44 (1.57)^3^ | ^4^ |

Using the formula:

N= z^2^ p (1-p)
 e^2^

Where the finite population correction is N(adjusted)= N.n
 N+n

Where z is the z-score for the normal distribution corresponding to the desired confidence level, p is the expected true proportion, e represents desired precision, n is the finite population size (the census population) and N is the estimated sample size requirement.

For sample size calculation for HCV community prevalence, we used prior conservative estimates of adult anti-HCV prevalence of 10% for Malawi based on data from the preceding 20 years from convenience samples ranging from 0.5% to 10% from a systematic review.^1^ To achieve a desired a precision (half-width) of 1.5% with 95% confidence intervals, the estimated sample size requirement was 1537.

To estimate sample size requirements to detect a significant difference between liver stiffness measurements in patients with hepatitis C and community control populations, we used the best available data for reference measurements in healthy populations, a systematic review of 26 cohort studies. For people living with hepatitis C infection in sub-Saharan Africa, the best available data reporting liver stiffness distribution was from a study of people who inject drugs from Tanzania. We calculated the sample size requirement using a two-sample means power analysis in Stata 17 (College Station, TX, USA). Using a two-sided t test with alpha of 0.05, power of 0.8 with mean and standard deviations based on the reference populations reported above, to detect a difference between the mean predicted liver stiffness of 4.6kPa in the normal population and 5.4kPa in people with hepatitis C, we estimated a sample size requirement of 63 in each group.

**Supplementary Appendix 2: Appendix: Systematic review of epidemiological data for hepatitis C seroprevalence in Southern Africa**

Literature searches:

Database: Pubmed (<https://pubmed.ncbi.nlm.nih.gov/>)
Date: 17 September 21
No language or date restrictions applied

| **Search** | **Search terms** | **Results** |
| --- | --- | --- |
| **1** | **Angola[Title/Abstract] or Botswana[Title/Abstract] or Eswatini[Title/Abstract] or Swaziland[Title/Abstract] Lesotho[Title/Abstract] or Malawi[Title/Abstract] or Mozambique[Title/Abstract] or Namibia[Title/Abstract] or (South Africa[Title/Abstract]) or Zambia[Title/Abstract] or Zimbabwe[Title/Abstract] or (southern Africa[Title/Abstract])** | **63,766** |
| **2** | **(hepatitis C[Title/Abstract]) or (anti-HCV[Title/Abstract]) or (HCV IgG[Title/Abstract]) or (HCV RNA[Title/Abstract])** | **83,569** |
| **3** | **#1 and #2** | **151** |

We conducted a search of hepatitis C virus (HCV) seroprevalence in the countries included in the southern region of the African Union, comprising: Angola, Botswana, Eswatini, Lesotho, Malawi, Mozambique, Namibia, South Africa, Zambia and Zimbabwe. We used terms of hepatitis C and synonyms of the diagnostic markers and no language or date restrictions were applied.

Of 151 identified studies, 100 were excluded due to having no epidemiological data on HCV, or studying countries outside the region of interest (Figure S1).

Groups considered to be at increased risk of HCV were studied in 11 articles comprising predominantly people who inject drugs. In 32 studies, a convenience sampling method was used to study HCV prevalence among attendees of HIV clinics, blood donors, antenatal populations attending for routine healthcare, HIV test centres or occupational samples. (Figure S1)

The full text of five articles was reviewed, of which three studied HCV prevalence among inpatients or those with established liver disease. The remaining two articles both studied general populations but did not provide a sampling method that would provide a representative sample. One study from a rural southern region of Malawi recruited 99 patients, but did not specify any details of a sampling method to facilitate an assessment of epidemiological representativeness.^5^ A second study from a rural region of Zimbabwe recruited patients to a study of schistosomiasis and retrospectively collected a subset (269/1545) of samples for HCV testing.^6^ However, this study did not report how the subset was selected and the overall sample was recruited by participation at a community meeting- no representative selection method was described.^7^

No studies that provided an estimation of HCV seroprevalence (antibody or HCV RNA) in the general population of southern Africa using a representative sampling method was identified.

**Figure S1: Flow chart of searches of hepatitis C epidemiology in southern Africa**

Exclusion:
3 Inpatient studies/ liver disease
2 no sampling framework

32 convenience sampling studies:
 14 HIV clinic
 13 Blood donors
 2 Antenatal
 2 HIV testing centre/ STD clinic
 1 Occupational

11 risk groups:
 6 people who inject drugs
 2 mixed populations
 1 haemodialysis
 1 men who have sex with men
 1 prison

No eligible studies identified

5 full text articles reviewed

100 excluded:

31 no HCV epidemiological data
 18 basic science/ sequencing/diagnostic evaluation
 17 review/editorial
 13 not southern Africa
 10 inpatients
 5 modelling study
 4 clinical trial/ treatment
 2 migrants

Basic science/ sequencing

Searches

Hepatitis C epidemiology in Southern Africa

n=151

**References:**

1. Taha TE, Rusie LK, Labrique A, et al. Seroprevalence for Hepatitis E and Other Viral Hepatitides among Diverse Populations, Malawi. *Emerg Infect Dis* 2015; **21**(7): 1174-82.

2. Kallestrup P, Zinyama R, Gomo E, et al. Low prevalence of hepatitis C virus antibodies in HIV-endemic area of Zimbabwe support sexual transmission as the major route of HIV transmission in Africa. *Aids* 2003; **17**(9): 1400-2.

3. Kallestrup P, Zinyama R, Gomo E, et al. Schistosomiasis and HIV-1 Infection in Rural Zimbabwe: Implications of Coinfection for Excretion of Eggs. *The Journal of Infectious Diseases* 2005; **191**(8): 1311-20.

**Studies of risk groups:**

1. Cassidy MJ, Jankelson D, Becker M, Dunne T, Walzl G, Moosa MR. The prevalence of antibodies to hepatitis C virus at two haemodialysis units in South Africa. *S Afr Med J* 1995; **85**(10): 996-8.

2. Chimphambano C, Komolafe I, Muula A. Prevalence of HIV, HepBsAg and Hep C antibodies among inmates in Chichiri prison, Blantyre, Malawi. *Malawi Med J* 2007; **19**(3): 107-10.

3. Meel R, Essop MR. Striking increase in the incidence of infective endocarditis associated with recreational drug abuse in urban South Africa. *S Afr Med J* 2018; **108**(7): 585-9.

4. Morgan N, Daniels W, Subramaney U. A prospective observational study of heroin users in Johannesburg, South Africa: Assessing psychiatric comorbidities and treatment outcomes. *Compr Psychiatry* 2019; **95**: 152137.

5. Prabdial-Sing N, Gaelejwe L, Makhathini L, et al. The performance of hepatitis C virus (HCV) antibody point-of-care tests on oral fluid or whole blood and dried blood spot testing for HCV serology and viral load among individuals at higher risk for HCV in South Africa. *Health Sci Rep* 2021; **4**(1): e229.

6. Scheibe A, Young K, Moses L, et al. Understanding hepatitis B, hepatitis C and HIV among people who inject drugs in South Africa: findings from a three-city cross-sectional survey. *Harm Reduct J* 2019; **16**(1): 28.

7. Scheibe A, Young K, Versfeld A, et al. Hepatitis B, hepatitis C and HIV prevalence and related sexual and substance use risk practices among key populations who access HIV prevention, treatment and related services in South Africa: findings from a seven-city cross-sectional survey (2017). *BMC Infect Dis* 2020; **20**(1): 655.

8. Semá Baltazar C, Boothe M, Kellogg T, et al. Prevalence and risk factors associated with HIV/hepatitis B and HIV/hepatitis C co-infections among people who inject drugs in Mozambique. *BMC Public Health* 2020; **20**(1): 851.

9. Semá Baltazar C, Horth R, Boothe M, et al. High prevalence of HIV, HBsAg and anti-HCV positivity among people who injected drugs: results of the first bio-behavioral survey using respondent-driven sampling in two urban areas in Mozambique. *BMC Infect Dis* 2019; **19**(1): 1022.

10. Semá Baltazar C, Kellogg TA, Boothe M, et al. Prevalence of HIV, viral hepatitis B/C and tuberculosis and treatment outcomes among people who use drugs: Results from the implementation of the first drop-in-center in Mozambique. *Int J Drug Policy* 2021; **90**: 103095.

11. Semugoma NP, Rebe K, Sonderup MW, et al. Hepatitis C: A South African literature review and results from a burden of disease study among a cohort of drug-using men who have sex with men in Cape Town, South Africa. *S Afr Med J* 2017; **107**(12): 1116-20.

**Convenience sampling studies:**

1. Andreotti M, Pirillo MF, Liotta G, et al. The impact of HBV or HCV infection in a cohort of HIV-infected pregnant women receiving a nevirapine-based antiretroviral regimen in Malawi. *BMC Infect Dis* 2014; **14**: 180.

2. Barth RE, Huijgen Q, Tempelman HA, Mudrikova T, Wensing AM, Hoepelman AI. Presence of occult HBV, but near absence of active HBV and HCV infections in people infected with HIV in rural South Africa. *J Med Virol* 2011; **83**(6): 929-34.

3. Candotti D, Mundy C, Kadewele G, Nkhoma W, Bates I, Allain JP. Serological and molecular screening for viruses in blood donors from Ntcheu, Malawi: high prevalence of HIV-1 subtype C and of markers of hepatitis B and C viruses. *J Med Virol* 2001; **65**(1): 1-5.

4. Casteling A, Song E, Sim J, et al. GB virus C prevalence in blood donors and high risk groups for parenterally transmitted agents from Gauteng, South Africa. *J Med Virol* 1998; **55**(2): 103-8.

5. Chasela CS, Wall P, Drobeniuc J, et al. Prevalence of hepatitis C virus infection among human immunodeficiency virus-1-infected pregnant women in Malawi: the BAN study. *J Clin Virol* 2012; **54**(4): 318-20.

6. Coffie PA, Egger M, Vinikoor MJ, et al. Trends in hepatitis B virus testing practices and management in HIV clinics across sub-Saharan Africa. *BMC Infect Dis* 2017; **17**(Suppl 1): 706.

7. Cunha L, Plouzeau C, Ingrand P, et al. Use of replacement blood donors to study the epidemiology of major blood-borne viruses in the general population of Maputo, Mozambique. *J Med Virol* 2007; **79**(12): 1832-40.

8. Demir M, Phiri S, Kaiser R, et al. HIV/Hepatitis C Virus Co-infection among Adults Beginning Antiretroviral Therapy, Malawi. *Emerg Infect Dis* 2016; **22**(11): 2018-20.

9. Ellis LA, Brown D, Conradie JD, et al. Prevalence of hepatitis C in South Africa: detection of anti-HCV in recent and stored serum. *J Med Virol* 1990; **32**(4): 249-51.

10. Fang CT, Field SP, Busch MP, Heyns Adu P. Human immunodeficiency virus-1 and hepatitis C virus RNA among South African blood donors: estimation of residual transfusion risk and yield of nucleic acid testing. *Vox Sang* 2003; **85**(1): 9-19.

11. Gogela NA, Sonderup MW, Rebe K, Chivese T, Spearman CW. Hepatitis C prevalence in HIV-infected heterosexual men and men who have sex with men. *S Afr Med J* 2018; **108**(7): 568-72.

12. Hoffmann CJ, Dayal D, Cheyip M, et al. Prevalence and associations with hepatitis B and hepatitis C infection among HIV-infected adults in South Africa. *Int J STD AIDS* 2012; **23**(10): e10-3.

13. Kapembwa KC, Goldman JD, Lakhi S, et al. HIV, Hepatitis B, and Hepatitis C in Zambia. *J Glob Infect Dis* 2011; **3**(3): 269-74.

14. Kurira P, Ndhlovu CE, Gomo ZA. Hepatitis B and C infection at a large public sector hospital clinic: is it a burden? *Cent Afr J Med* 2014; **60**(9-12): 56-62.

15. Loarec A, Carnimeo V, Molfino L, et al. Extremely low hepatitis C prevalence among HIV co-infected individuals in four countries in sub-Saharan Africa. *Aids* 2019; **33**(2): 353-5.

16. Lodenyo H, Schoub B, Ally R, Kairu S, Segal I. Hepatitis B and C virus infections and liver function in AIDS patients at Chris Hani Baragwanath Hospital, Johannesburg. *East Afr Med J* 2000; **77**(1): 13-5.

17. M'Baya B, Jumbe V, Samuel V, M'Bwana R, Mangani C. Seroprevalence and trends in transfusion transmissible infections among voluntary non-remunerated blood donors at the Malawi Blood Transfusion Service-a time trend study. *Malawi Med J* 2019; **31**(2): 118-25.

18. Madzime S, William MA, Mohamed K, et al. Seroprevalence of hepatitis C virus infection among indigent urban pregnant women in Zimbabwe. *Cent Afr J Med* 2000; **46**(1): 1-4.

19. Maida MJ, Daly CC, Hoffman I, Cohen MS, Kumwenda M, Vernazza PL. Prevalence of hepatitis C infection in Malawi and lack of association with sexually transmitted diseases. *Eur J Epidemiol* 2000; **16**(12): 1183-4.

20. Mavenyengwa RT, Mukesi M, Chipare I, Shoombe E. Prevalence of human immunodeficiency virus, syphilis, hepatitis B and C in blood donations in Namibia. *BMC Public Health* 2014; **14**: 424.

21. Mvere D, Constantine NT, Katsawde E, Tobaiwa O, Dambire S, Corcoran P. Rapid and simple hepatitis assays: encouraging results from a blood donor population in Zimbabwe. *Bull World Health Organ* 1996; **74**(1): 19-24.

22. Parboosing R, Paruk I, Lalloo UG. Hepatitis C virus seropositivity in a South African Cohort of HIV co-infected, ARV naïve patients is associated with renal insufficiency and increased mortality. *J Med Virol* 2008; **80**(9): 1530-6.

23. Schoub BD, Johnson S, McAnerney JM, Blackburn NK. The role of sexual transmission in the epidemiology of hepatitis C virus in black South Africans. *Trans R Soc Trop Med Hyg* 1992; **86**(4): 431-3.

24. Stokx J, Gillet P, De Weggheleire A, et al. Seroprevalence of transfusion-transmissible infections and evaluation of the pre-donation screening performance at the Provincial Hospital of Tete, Mozambique. *BMC Infect Dis* 2011; **11**: 141.

25. Sutcliffe S, Taha TE, Kumwenda NI, Taylor E, Liomba GN. HIV-1 prevalence and herpes simplex virus 2, hepatitis C virus, and hepatitis B virus infections among male workers at a sugar estate in Malawi. *J Acquir Immune Defic Syndr* 2002; **31**(1): 90-7.

26. Tamandjou Tchuem C, Cotton MF, Nel E, et al. Viral hepatitis B and C in HIV-exposed South African infants. *BMC Pediatr* 2020; **20**(1): 563.

27. Tremeau-Bravard A, Ogbukagu IC, Ticao CJ, Abubakar JJ. Seroprevalence of hepatitis B and C infection among the HIV-positive population in Abuja, Nigeria. *Afr Health Sci* 2012; **12**(3): 312-7.

28. Vardas E, Sitas F, Seidel K, Casteling A, Sim J. Prevalence of hepatitis C virus antibodies and genotypes in asymptomatic, first-time blood donors in Namibia. *Bull World Health Organ* 1999; **77**(12): 965-72.

29. Vermeulen M, Swanevelder R, Chowdhury D, et al. Use of Blood Donor Screening to Monitor Prevalence of HIV and Hepatitis B and C Viruses, South Africa. *Emerg Infect Dis* 2017; **23**(9): 1560-3.

30. Vinikoor MJ, Mulenga L, Siyunda A, et al. Association between hepatitis B co-infection and elevated liver stiffness among HIV-infected adults in Lusaka, Zambia. *Trop Med Int Health* 2016; **21**(11): 1435-41.

31. Wandeler G, Mulenga L, Hobbins M, et al. Absence of Active Hepatitis C Virus Infection in Human Immunodeficiency Virus Clinics in Zambia and Mozambique. *Open Forum Infect Dis* 2016; **3**(2): ofw049.

32. Wolff C, Hörnschemeyer D, Skurtopulos M, et al. [Molecular biological screening of viruses important to transfusion medicine]. *Beitr Infusionsther Transfusionsmed* 1994; **32**: 102-9.

**Supplementary Appendix 3: Comparison of census and serosurvey age and sex distribution**

**
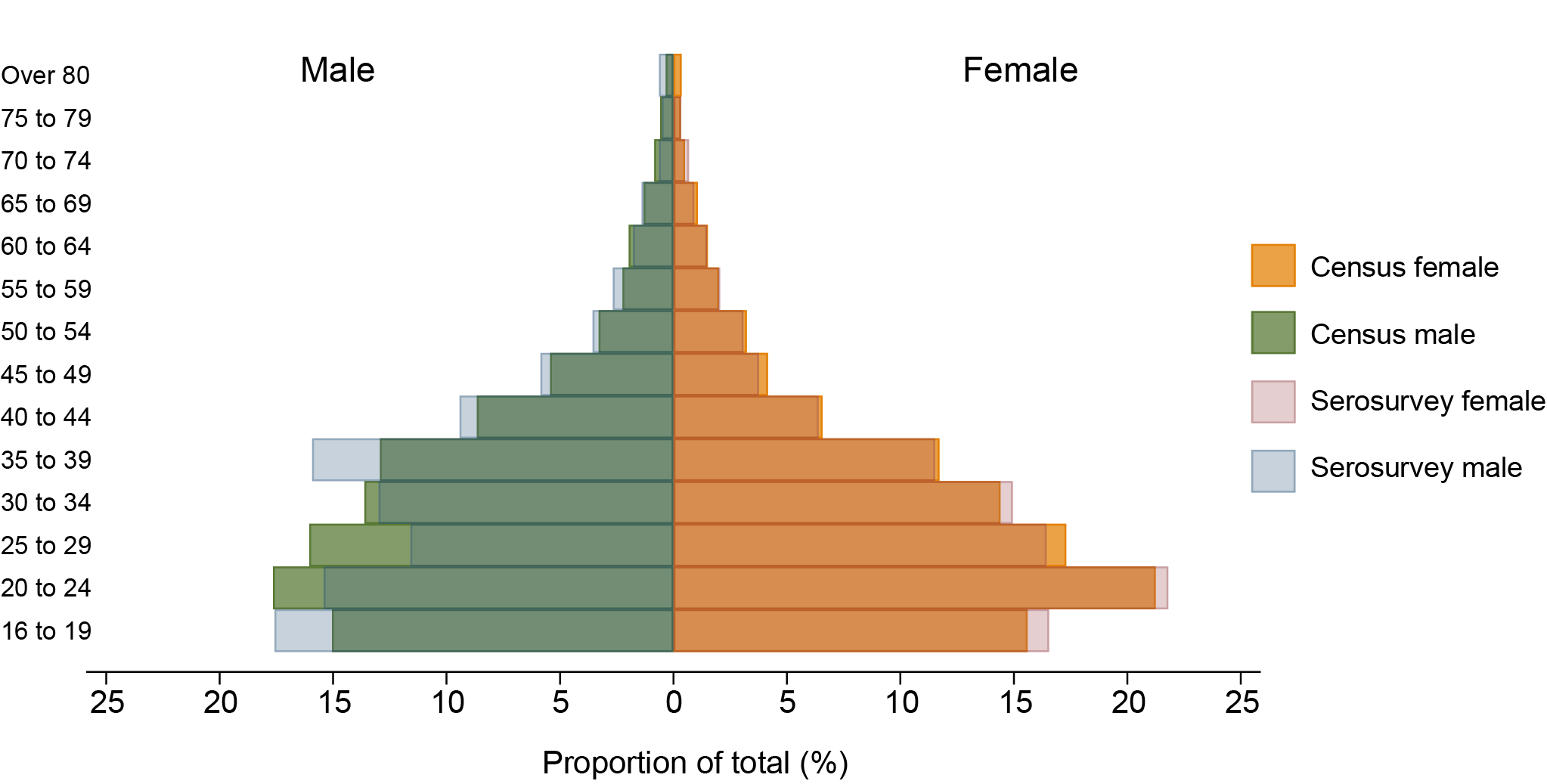
**

**Supplementary Appendix 4: Characteristics of HCV RNA positive hospital patients with cirrhosis or HCC recruited from Queen Elizabeth Central Hospital**

| **Characteristic**  **N(%) or median (IQR)** | **HCV RNA positive patients**  **(n=7)** |
| --- | --- |
| Age, years | 69 (64, 73) |
| Sex, male (%) | 3 (43) |
| Presenting symptoms |  |
| Abdominal pain | 4 (57) |
| Abdominal swelling | 3 (43) |
| Leg swelling | 2 (29) |
| Shortness of breath | 1 (14) |
| Weight loss | 1 (14) |
| Duration of symptoms, months | 4.0 (2.0, 6.6) |
| Signs of chronic liver disease | 6 (86) |
| Cachexia | 5 (71) |
| Ascites | 5 (71) |
| Peripheral oedema | 3 (43) |
| Median liver stiffness, kPa | >75 (27.2, >75) |
| Body mass index, kg/m^2^ | 21 (20, 24) |
| HCC characteristics (among patients with HCC, n=4) |  |
| Largest lesion size (cm) | 8.7 (6.1, 12.1) |
| Portal vein compression or invasion | 4 (100) |

**Supplementary Appendix 5: Association between HCV Ab/Ag ELISA sample to cut-off (S/CO) ratio and HCV RNA and line immunoassay status^a^**

**
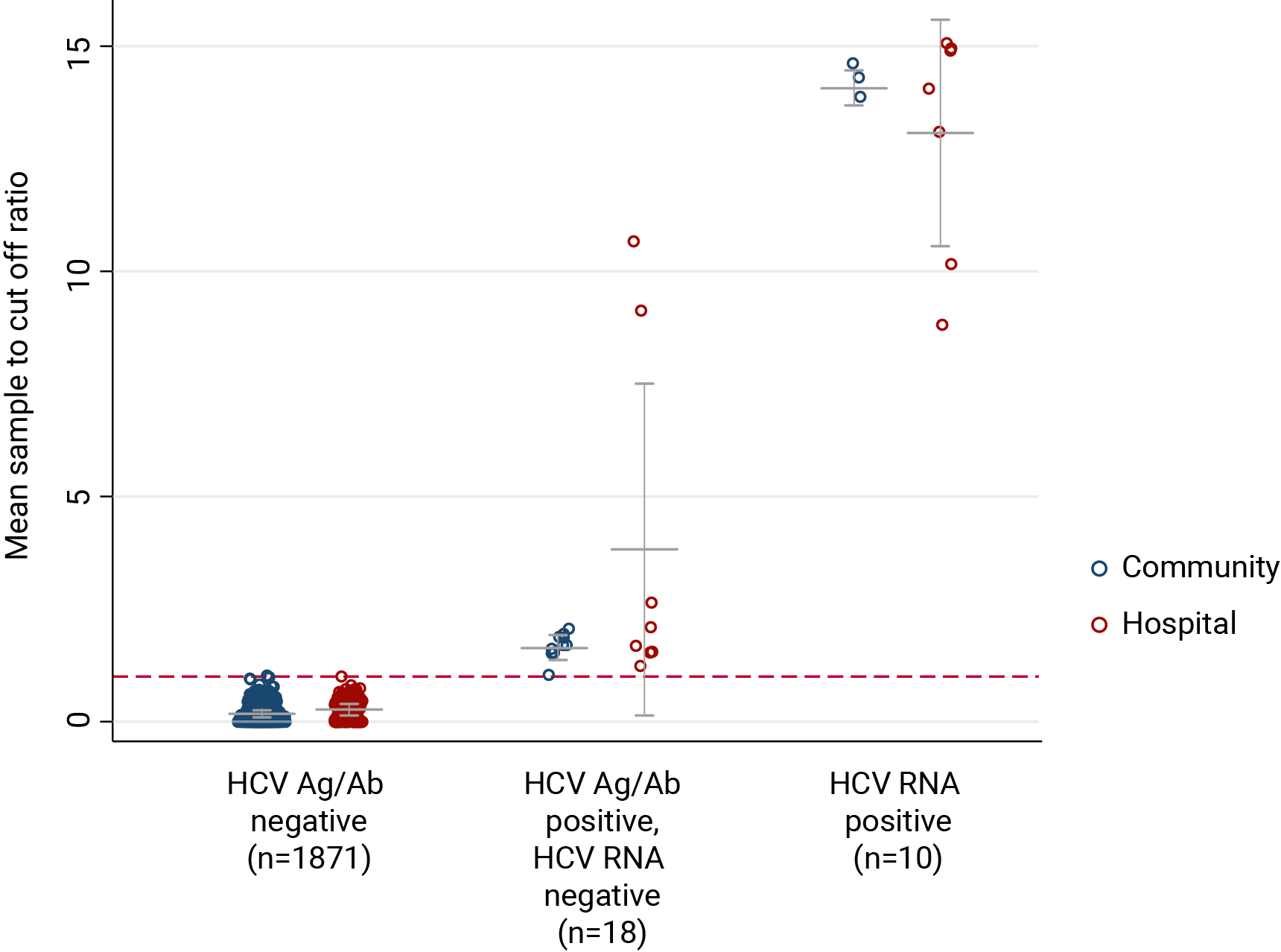
**

^a^ S/CO >1 is interpreted as positive (red dotted line) as per manufacturer’s instructions. Grey horizontal lines show mean and one standard deviation.

**References**

1. Stockdale AJ, Mitambo C, Everett D, Geretti AM, Gordon MA. Epidemiology of hepatitis B, C and D in Malawi: systematic review. *BMC Infect Dis* 2018; **18**(1): 516.

2. Bazerbachi F, Haffar S, Wang Z, et al. Range of Normal Liver Stiffness and Factors Associated With Increased Stiffness Measurements in Apparently Healthy Individuals. *Clin Gastroenterol Hepatol* 2019; **17**(1): 54-64.e1.

3. Wan X, Wang W, Liu J, Tong T. Estimating the sample mean and standard deviation from the sample size, median, range and/or interquartile range. *BMC Med Res Methodol* 2014; **14**(1): 135.

4. Mohamed Z, Rwegasha J, Kim JU, et al. The hepatitis C cascade of care in people who inject drugs in Dar es Salaam, Tanzania. *J Viral Hepat* 2018; **25**(12): 1438-45.

5. Taha TE, Rusie LK, Labrique A, et al. Seroprevalence for Hepatitis E and Other Viral Hepatitides among Diverse Populations, Malawi. *Emerg Infect Dis* 2015; **21**(7): 1174-82.

6. Kallestrup P, Zinyama R, Gomo E, et al. Low prevalence of hepatitis C virus antibodies in HIV-endemic area of Zimbabwe support sexual transmission as the major route of HIV transmission in Africa. *Aids* 2003; **17**(9): 1400-2.

7. Kallestrup P, Zinyama R, Gomo E, et al. Schistosomiasis and HIV-1 Infection in Rural Zimbabwe: Implications of Coinfection for Excretion of Eggs. *The Journal of Infectious Diseases* 2005; **191**(8): 1311-20.
